# Correlates of pedometer use: Results from a community-based physical activity intervention trial (10,000 Steps Rockhampton)

**DOI:** 10.1186/1479-5868-4-31

**Published:** 2007-07-27

**Authors:** Elizabeth G Eakin, Kerry Mummery, Marina M Reeves, Sheleigh P Lawler, Grant Schofield, Alison J Marshall, Wendy J Brown

**Affiliations:** 1 Cancer Prevention Research Centre, School of Population Health, The University of Queensland, Brisbane, Australia; 2 School of Human Movement Studies, The University of Queensland, Brisbane, Australia; 3 School of Health and Human Performance, Central Queensland University, Rockhampton, Australia; 4 Centre for Physical Activity and Nutrition Research, Auckland University of Technology, Auckland, New Zealand

## Abstract

**Background:**

Pedometers have become common place in physical activity promotion, yet little information exists on who is using them. The multi-strategy, community-based 10,000 Steps Rockhampton physical activity intervention trial provided an opportunity to examine correlates of pedometer use at the population level.

**Methods:**

Pedometer use was promoted across all intervention strategies including: local media, pedometer loan schemes through general practice, other health professionals and libraries, direct mail posted to dog owners, walking trail signage, and workplace competitions. Data on pedometer use were collected during the 2-year follow-up telephone interviews from random population samples in Rockhampton, Australia, and a matched comparison community (Mackay). Logistic regression analyses were used to determine the independent influence of interpersonal characteristics and program exposure variables on pedometer use.

**Results:**

Data from 2478 participants indicated that 18.1% of Rockhampton and 5.6% of Mackay participants used a pedometer in the previous 18-months. Rockhampton pedometer users (n = 222) were more likely to be female (OR = 1.59, 95% CI: 1.11, 2.23), aged 45 or older (OR = 1.69, 95% CI: 1.16, 2.46) and to have higher levels of education (university degree OR = 4.23, 95% CI: 1.86, 9.6). Respondents with a BMI > 30 were more likely to report using a pedometer (OR = 1.68, 95% CI: 1.11, 2.54) than those in the healthy weight range. Compared with those in full-time paid work, respondents in 'home duties' were significantly less likely to report pedometer use (OR = 0.18, 95% CI: 0.06, 0.53). Exposure to individual program components, in particular seeing 10,000 Steps street signage and walking trails or visiting the website, was also significantly associated with greater pedometer use.

**Conclusion:**

Pedometer use varies between population subgroups, and alternate strategies need to be investigated to engage men, people with lower levels of education and those in full-time 'home duties', when using pedometers in community-based physical activity promotion initiatives.

## Background

The evidence supporting the role of physical activity in the prevention and management of a wide range of chronic diseases is overwhelming [1,2]. However, while the health benefits of physical activity are well established, in Australia, as in other industrialised countries, nearly one half of the population do not meet the recommended guidelines for physical activity [3,4]. Australian National Physical Activity Guidelines recommend a minimum of 150 minutes of moderate-intensity activity a week, and being active on most days of the week [5]. Given increasing trends in overweight and obesity, interventions to increase population levels of physical activity are now at the forefront of health promotion efforts.

In recent years, the pedometer (step counter), initially used as a physical activity measurement tool [6], has become widely adopted as a physical activity intervention tool [7,8]. Although generally based on small samples, interventions incorporating pedometers have been shown to be effective in improving physical activity levels (and associated disease markers) in both controlled trials in clinical samples [9-13] and, more recently, in settings such as work sites and churches [7,8]. However, much less is known about the use of pedometers in the context of community-based trials, particularly the characteristics of pedometer users.

This paper describes the correlates of pedometer use in an Australian population-based sample that spanned two communities. In one community, pedometers were promoted as part of a multi-component, community-based physical activity intervention (10,000 Steps Rockhampton), while the other acted as a matched comparison community that did not receive the intervention. Physical activity changes resulting from the trial have been described in detail elsewhere. In brief, the primary outcome of this intervention was a 5% increase in the proportion of 'active' women in the intervention community compared to a downward trend in the comparison community [14]. In this paper, we examine associations between intrapersonal characteristics (i.e. demographic and health variables) and program exposure variables with pedometer use from both the intervention and comparison communities.

## Methods

The data presented here are from the follow-up survey of more than 2000 adults who participated in a two-year, multi-strategy, community-based physical activity intervention (10,000 Steps Rockhampton). The methods and primary outcomes of the 10,000 Steps Rockhampton project have been described previously [14,15]. Cross-sectional surveys were conducted at baseline (August – September 2001) and follow-up (August – September 2003) from computer-assisted telephone interview (CATI) surveys in Rockhampton, Queensland (the intervention community) and a matched comparison community (Mackay, Queensland). Rockhampton is situated approximately 700 km north of the state capital, with a population of 60,000, while Mackay has a population of 75,000 and is located 400 km to the north of Rockhampton. The samples were drawn at random from the regularly updated electronic database of telephone numbers in Rockhampton and Mackay. The study protocol was approved by the Human Ethics Research Review Panel at Central Queensland University.

### Promotion of Pedometers through the 10,000 Steps Intervention

The 10,000 Steps Rockhampton intervention involved four key strategies, all of which promoted the use of pedometers and step counting; – 1) a media campaign; 2) engaging general practitioners (GPs) and other health professionals in promoting physical activity [16,17]; 3) worksite physical activity promotion; and 4) working with local government on environmental supports (i.e. signage and walking trails [18]).

The use of pedometers as an individual self-monitoring and goal setting instrument was the fundamental component of the overall media campaign. In addition to the overarching theme of '10,000 Steps a Day', a secondary theme, 'Every Step Counts' was used to emphasise the 'accumulation' aspect of current PA guidelines and to encourage people to find ways of increasing daily steps, even if they did not reach the 10,000 steps target. Approximately 2500 pedometers and logbooks were made available for purchase through the local project office, the project web-site and from local pharmacies (cost per pedometer approximately $40 AUD). Limited numbers (about 500) were available for loan from local libraries and GP/health professionals. Five large workplaces also made approximately 2000 pedometers available to their employees. Rockhampton residents were also able to purchase pedometers from sports stores and the local office of the National Heart Foundation.

### Measures

As part of the follow-up survey in 2003, respondents were asked "Have you used a pedometer to count your steps in the last 18 months?". Additional questions were asked to those who reported using a pedometer in the previous 18 months – overall duration of use (days, weeks, months); frequency of use (daily, weekly or occasionally); average number of steps; helpfulness of the pedometer in increasing physical activity; and place of purchase. Other data collected from all respondents included demographics (age, sex, education level, employment status, etc), medical history (number of chronic diseases, height, weight, etc) and physical activity level. Physical activity was assessed using the Active Australia Survey [5]. Total physical activity scores were used to categorise individuals, based on current public health recommendations for physical activity [19], into: sufficiently active (150 minutes or more of moderate-intensity physical activity over 5 or more days/week), insufficiently active (1–149 minutes of at least moderate-intensity activity and/or < 5 days/week), inactive (zero minutes of physical activity) [20].

Respondents from Rockhampton were also asked questions relating to awareness of the 10,000 Steps Rockhampton project and its components. These included whether they had received any physical activity advice from their GP, whether they had visited the 10,000 Steps Website, whether they remembered receiving materials from the 10,000 Steps project in the mail, or had seen 10,000 Steps street signage and walking trails, and whether they were aware of a "million steps" promotion that had been conducted during the 10,000 Steps program in conjunction with an Australian milk producer.

### Statistical Analysis

Data were analysed using SPSS for Windows (Version 13.0, 2004, SPSS Inc., Chicago, USA) statistical software package. Descriptive statistics (counts and percentages) were calculated. At the bivariate level, chi-squared tests were used to explore the association between pedometer use and demographic, health, physical activity and program exposure variables. Variables shown to be significantly (or clinically meaningfully) associated at the bivariate level were included in logistic regression models to determine their independent influences on pedometer use. Step one assessed the influence of intrapersonal characteristics on pedometer use (Model 1) and step two assessed the additional independent influence of program exposure on pedometer use (Model 2). Results are expressed as odds ratios with 95% confidence intervals. Statistical significance was set at p < 0.05 (two-tailed).

## Results

Data were collected in 2003 from 2478 participants (overall response rate 47.3%: Rockhampton n = 1242, Mackay n = 1236; mean age 45 ± 17 years, range 18–94 years). Demographic characteristics of the participants are shown in Table 1. There were no significant differences in these characteristics for respondents from the two communities, and the age and employment distributions of the sample were not significantly different from the 2001 census data for these areas (data not shown).

**Table 1:** Demographic characteristics of the total sample of participants in 2003 (n = 2478)

**Characteristic**	**n**	**%**
**Age Group**		
18 – 44 years	1357	54.8
45 years and over	1121	45.2
**Gender**		
Male	1233	49.8
Female	1245	50.2
**Location**		
Mackay	1236	49.9
Rockhampton	1242	50.1
**Highest Education Level**		
Primary School	210	8.5
High School to Year 10	611	24.7
High School to Year 12	476	19.2
Technical Cert/Dip	700	28.2
University degree	428	17.3
Missing	53	2.1
**Employment**		
Full-time	1022	41.2
Part-time/Casual	488	19.7
Home Duties	209	8.4
Student/Unemployed/Unable to work	155	6.3
Retired	454	18.3
Other	110	4.4
Missing	40	1.6
**Household Income (per week)**		
Less than $300	242	9.8
$300 – $599	380	15.3
$600 – $999	437	17.6
$1000 or more	753	30.4
Missing	666	26.9
**Living Situation**		
Alone	367	14.8
With other(s)	2064	83.3
Missing	47	1.9
Smoking Status		
Never smoker	1228	49.6
Ex-smoker	738	29.8
Current smoker	481	19.4
Missing	31	1.3
**BMI (kg/m2)**		
< 18.5	55	2.2
18.5 – 24.9	978	39.5
25 – 29.9	807	32.6
≥ 30	478	19.3
Missing	160	6.5
**Physical Activity Level**		
Sedentary	437	17.6
Insufficiently active	934	37.7
Sufficiently active	1008	40.7
Missing	99	4.0
**Number of Chronic Diseases**		
None	1750	70.6
1	487	19.7
2	158	6.4
3 or more	51	2.1
Missing	32	1.3

BMI, body mass index

### Community-based pedometer use

Overall, 291 (11.8%) participants reported using a pedometer in the previous 18 month period, with significantly greater pedometer use in Rockhampton (18.1%) than in Mackay (5.6%, p < 0.001). Though not statistically significant, among those who reported using a pedometer, the odds of currently wearing the pedometer on a daily basis was also higher in Rockhampton (OR = 2.24, 95% CI: 0.96, 5.23; p = 0.06). However, 82% of the 291 respondents who reported using a pedometer at some point during the past 18 months were no longer using the pedometer at the time of the follow-up survey. Overall duration of pedometer use varied from a few days to more than one year in both communities, with 65% of respondents reporting use of a pedometer for at least one month. There was no significant difference between communities in terms of reported duration of pedometer use or average daily steps. Just over half (54%) of the pedometer users rated the pedometer as helpful in increasing their physical activity level. Half (50%) of all pedometer users reported buying a pedometer and 47% borrowed one (from a GP, the library, workplace, or family and friends).

### Intrapersonal characteristics and pedometer use

As promotion of pedometers was one of the main components of the intervention in Rockhampton, but did not occur in the comparison community (Mackay), logistic regression models assessing intrapersonal characteristics of pedometer users were performed separately for Rockhampton and Mackay. Among the Rockhampton respondents (n = 1242), bivariate analyses found that pedometer use in the last 18 months differed significantly by age group (p = 0.04), education level (p = 0.003), employment status (p = 0.005), household income (p = 0.033) and physical activity level (p = 0.006). Although not statistically significant, there were also clinically meaningful associations between pedometer use and gender (p = 0.120), body mass index (BMI) (p = 0.182) and number of chronic conditions (p = 0.497) (Table 2). Living situation and smoking status were not significantly associated with pedometer use.

**Table 2:** Unadjusted and adjusted odds ratios and 95% CIs for pedometer use among Rockhampton residents in 2003

	Used a pedometer n (%)	Unadjusted OR (95% CI)	**Model 1 **Adjusted OR^1 ^(95% CI)	p-value^1^	**Model 2 **Adjusted OR^2 ^(95% CI)	p-value^2^
**Age Group**						
18 – 44 years	110 (16.1)	1.00	1.00		1.00	
45 years and over	112 (20.7)	1.36 (1.01, 1.82)	1.69 (1.16, 2.46)	0.006	1.64 (1.06, 2.52)	0.026
**Gender**						
Male	100 (16.4)	1.00	1.00		1.00	0.335
Female	122 (19.8)	1.26 (0.94, 1.69)	1.59 (1.13, 2.23)	0.007	1.21 (0.82, 1.78)	
**Employment**						
Full -time	104 (20.4)	1.00	1.00		1.00	0.093
Part time/Casual	50 (20.8)	1.03 (0.70, 1.50)	1.07 (0.70, 1.65)	0.752	1.08 (0.66, 1.76)	
Home Duties	5 (4.6)	0.19 (0.08, 0.48)	0.18 (0.06, 0.53)	0.002	0.21 (0.07, 0.63)	
Student/unemployed/unable to work	16 (17.8)	0.84 (0.47, 1.51)	0.95 (0.50, 1.84)	0.889	1.04 (0.49, 2.19)	
Retired	38 (17.0)	0.80 (0.53, 1.20)	0.99 (0.58, 1.67)	0.963	1.03 (0.57, 1.85)	
Other	6 (14.0)	0.63 (0.26, 1.54)	0.63 (0.25, 1.60)	0.330	0.62 (0.21, 1.81)	
**Education Level**						
Primary School	12 (11.1)	1.00	1.00		1.00	0.082
High School to Year 10	46 (14.2)	1.32 (0.67, 2.59)	1.78 (0.81, 3.95)	0.153	1.47 (0.63, 3.42)	
High School to Year 12	43 (18.1)	1.77 (0.89, 3.52)	2.70 (1.16, 6.28)	0.021	1.92 (0.77, 4.78)	
Technical Cert/Dip	61 (18.9)	1.86 (0.96, 3.61)	2.52 (1.13, 5.59)	0.023	2.02 (0.87, 4.71)	
University degree	58 (27.1)	2.97 (1.52, 5.82)	4.23 (1.86, 9.66)	0.001	2.88 (1.19, 6.97)	
**BMI Category**						
18.5 – 24.9 kg/m2	85 (17.2)	1.00	1.00		1.00	0.132
< 18.5 kg/m2	2 (7.4)	0.39 (0.09, 1.66)	0.44 (0.10, 1.99)	0.288	0.28 (0.03, 2.27)	
25 – 29.9 kg/m^2^	69 (17.7)	1.04 (0.73, 1.47)	1.16 (0.80, 1.69)	0.426	1.30 (0.85, 1.99)	
≥ 30 kg/m^2^	54 (22.0)	1.36 (0.93, 1.99)	1.68 (1.11, 2.54)	0.017	1.60 (0.99, 2.58)	
**Physical Activity Level**						
Sedentary	33 (15.3)	1.00	1.00		1.00	0.079
Insufficiently active	70 (15.3)	0.99 (0.63, 1.56)	0.90 (0.55, 1.45)	0.653	0.75 (0.43, 1.30)	
Sufficiently active	114 (22.5)	1.60 (1.05, 2.45)	1.50 (0.95, 2.38)	0.084	1.19 (0.70, 2.02)	
**Number of Chronic Diseases**						
None	156 (18.0)	1.00	1.00	0.398	1.00	0.320
1	51 (20.0)	1.14 (0.80, 1.62)	1.03 (0.69, 1.54)		0.93 (0.59, 1.46)	
2	12 (15.4)	0.83 (0.44, 1.57)	0.79 (0.37, 1.66)		0.65 (0.29, 1.44)	
3 or more	2 (8.7)	0.43 (0.10, 1.87)	0.19 (0.02, 1.49)		0.17 (0.02, 1.46)	
**Program Exposure**						
Physical activity advice from GP						
No	128 (17.3)	1.00			1.00	0.095
Yes	68 (24.7)	1.57 (1.13, 2.19)			1.43 (0.94, 2.17)	
Awareness of 'Million Steps' milk promotion						
No	134 (15.7)	1.00			1.00	
Yes	86 (28.0)	2.08 (1.53, 2.84)			1.51 (1.02, 2.23)	0.038
Visited 10,000 Steps Website						
No	202 (17.9)	1.00			1.00	
Yes	18 (52.9)	5.15 (2.58, 10.28)			2.63 (1.15, 6.05)	0.022
Remember receiving materials from 10,000 Steps in the mail						
No	148 (16.3)	1.00			1.00	
Yes	72 (29.0)	2.10 (1.52, 2.91)			1.54 (1.03, 2.31)	0.037
Seen 10,000 Steps street signage & walking trails						
No	81 (12.7)	1.00			1.00	
Yes	139 (26.7)	2.51 (1.85, 3.40)			2.80 (1.91, 4.10)	< 0.001

Abbreviations: OR, odds ratio; CI, confidence interval; BMI, body mass index; GP, general practitioner.

^1 ^Adjusted for all demographic variables shown in the table (n = 1107)

^2 ^Adjusted for all demographic and exposure variables shown in the table (n = 865)

Variables with a significant bivariate relationship with pedometer use, and those that appeared to have a meaningful, although not statistically significant, relationship, were considered in a multivariable logistic regression model to determine their independent influences on pedometer use among Rockhampton residents (see Table 2, Model 1). Household income was excluded from the final multivariable model because there was a large amount of missing data for this variable. After mutual adjustment for all variables included in the first model, age, gender, employment status, education level and BMI category remained as significant predictors of pedometer use (see Table 2).

The odds of using a pedometer were higher in women than in men and higher in respondents aged 45 years or more than in those aged under 45 years. The odds of using a pedometer increased with increasing years of education, with those in the 'university degree' category being more than four times more likely to report using a pedometer. The lowest odds for pedometer use were seen among those who reported 'home duties' as their main occupation. Respondents with a BMI in the obese category were significantly more likely to report using a pedometer. The associations between pedometer use and number of chronic conditions and physical activity, identified in the bivariate analyses, were attenuated and no longer significant after adjustment for the other factors in the model.

When the same variables were considered in a logistic regression model using data reported by the Mackay residents, physical activity level was the only variable to be significantly associated with pedometer use. The odds of using a pedometer were 2.1 (95%CI: 0.97, 4.53; p = 0.059) times higher in residents who were sufficiently active than in sedentary individuals.

### Program exposure and pedometer use

In bivariate analyses, all five program exposure variables were associated with pedometer use in Rockhampton (see Table 2). However, when these program exposure variables were included with the interpersonal characteristics in Model 2, the odds ratio for receipt of physical activity advice from a GP was attenuated and no longer significant. The strongest associations were for seeing the street signage/walking trails and visiting the website. The associations with interpersonal characteristics that were observed in Model 1 were also attenuated, with only age remaining statistically significant.

## Discussion

The 10,000 Steps Rockhampton project promoted physical activity and 'step counting' in a multi-strategy community-based intervention. This paper is one of the first to describe the factors related to pedometer use in the general population, as well as to examine the effect of a multi-strategy community-based intervention on pedometer use. While pedometer use was modest (approximately one fifth of respondents to the population-based survey in Rockhampton), it was significantly more than in the comparison community of Mackay (5.6%). From a public health perspective this difference is relevant as it equates to approximately 7,200 adult community residents (from the intervention community of approximately 60,000 residents) using a pedometer.

Our evaluation of factors associated with pedometer use was consistent with results from the overall trial [14,21], namely that women were the early adopters in terms of pedometer use and with regard to increases in physical activity. Pedometer use was also more likely among the employed and educated, suggesting that there is still considerable work to be done to improve pedometer use among those most in need (i.e., lower SES). However, it was encouraging that in Rockhampton, obese people were more likely to use pedometers. This finding is consistent with the results of the general practitioner strategy implemented as part of the 10,000 steps program, where respondents who were overweight or obese were more likely to report receiving physical activity advice from their GP [22]. In contrast, in the comparison community, physical activity (being sufficiently active) was the only factor associated with pedometer use, suggesting that without intervention, pedometer use is likely to be more prevalent among those who are already active.

There are limited studies against which to compare our findings; there is no published report describing the natural history of pedometer use in the general population, and only one study has evaluated pedometer use in the context of a community-based intervention trial. In a descriptive study using pedometers to determine the average step counts of a general population sample, Tudor- Locke and colleagues found that two-thirds of those who agreed to participate were overweight or obese; they were also more likely to be white, to have a higher education, and to have a higher household income [23].

Similar findings were reported by Craig et al. from the Canada on the Move initiative, a physical activity (walking) awareness and promotion campaign involving mass marketing messages and distribution of pedometers in cereal boxes [24]. This study reported that pedometer use was more likely among women and older people (44–64 years), as well as being associated with campaign awareness.

In our study, all the program exposure variables, with the exception of physical activity advice from the GP, were associated with an increased likelihood of pedometer use. While this suggests that the community-based physical activity intervention may have had an impact on pedometer use, the cross-sectional nature and resultant inability to infer causality should be noted. An alternative explanation is that those who had a pedometer may have been more likely to notice cues such as street signs and to engage with pedometer-related program strategies, such as visiting the website for more information or to log their steps.

Although they did not involve whole-of-community interventions, two other recent trials have evaluated the use of pedometers in specific community settings: workplaces and churches [7,8]. The successful implementation of pedometer-based intervention protocols in both these trials provides further support for the efficacy of pedometers for use in reaching large numbers of people for physical activity promotion.

The strengths of this study include the use of pedometers as part of a whole-of-community approach to physical activity promotion, as well as use of a population-based survey to evaluate pedometer use and related socio-demographic and health correlates. The study results help us to better understand pedometer use in various subgroups, and highlight the need to target specific population subgroups not reached by the initial 10,000 Steps intervention. The limitations include the low survey response rate and the cross-sectional nature of the data. The fact that the pedometer use questions were not asked in the baseline survey is also a limitation, as we are unable to say whether those who reported using a pedometer were 'new' pedometer users. The much higher rate of use in Rockhampton does however strongly suggest that the intervention was successful.

## Conclusion

The results of this study show that, when promoted as part of a multi-strategy, community-based intervention, pedometer use was greatest amongst women, older people, those with higher levels of education and obese people. Pedometer use was significantly less likely in those who reported 'home-duties' as their main occupation. Not surprisingly, greater program exposure was associated with significantly higher rates of pedometer use, even after adjustment for the demographic variables. The limited overall use of pedometers in the intervention community suggests that there is still much to be done to promote their use as part of population-based physical activity promotion efforts. Results of this study also suggest that efforts to promote pedometers will need to be mindful of those who are more difficult to reach with traditional health promotion efforts, including men, people with lower levels of education and those in 'home-duties'. Consideration should also be given to involving children in schools to broaden the reach of pedometers across the community.

## Declaration of Competing interests

The author(s) declare that they have no competing interests.

## Authors' contributions

WJB, EGE, KM and GS initiated and designed the study. MMR conducted the statistical analyses. EGE, MMR, SPL, WJB and ALM contributed to the writing of the manuscript. All authors read and approved the final manuscript.
